# RNA Editing, ADAR1, and the Innate Immune Response

**DOI:** 10.3390/genes8010041

**Published:** 2017-01-18

**Authors:** Qingde Wang, Xiaoni Li, Ruofan Qi, Timothy Billiar

**Affiliations:** Department of Surgery, University of Pittsburgh School of Medicine, Pittsburgh, PA 15213, USA; lix10@upmc.edu (X.L.); qir@upmc.edu (R.Q.)

**Keywords:** RNA editing, ADAR1, innate immune, RNA sensing

## Abstract

RNA editing, particularly A-to-I RNA editing, has been shown to play an essential role in mammalian embryonic development and tissue homeostasis, and is implicated in the pathogenesis of many diseases including skin pigmentation disorder, autoimmune and inflammatory tissue injury, neuron degeneration, and various malignancies. A-to-I RNA editing is carried out by a small group of enzymes, the adenosine deaminase acting on RNAs (ADARs). Only three members of this protein family, ADAR1–3, exist in mammalian cells. ADAR3 is a catalytically null enzyme and the most significant function of ADAR2 was found to be in editing on the neuron receptor GluR-B mRNA. ADAR1, however, has been shown to play more significant roles in biological and pathological conditions. Although there remains much that is not known about how ADAR1 regulates cellular function, recent findings point to regulation of the innate immune response as an important function of ADAR1. Without appropriate RNA editing by ADAR1, endogenous RNA transcripts stimulate cytosolic RNA sensing receptors and therefore activate the IFN-inducing signaling pathways. Overactivation of innate immune pathways can lead to tissue injury and dysfunction. However, obvious gaps in our knowledge persist as to how ADAR1 regulates innate immune responses through RNA editing. Here, we review critical findings from ADAR1 mechanistic studies focusing on its regulatory function in innate immune responses and identify some of the important unanswered questions in the field.

## 1. ADAR1 and RNA Editing

Following the pioneering discovery of Dr. Bass and colleagues that the conversion of adenosine to inosine was the basis underlying dsRNA-unwinding, and that this conversion was mediated by an adenosine deaminase [1,2], mammalian ADAR1 (originally called double-stranded RNA adenosine deaminase, or DRADA) was first purified from bovine liver nuclear extract [3]. Its cDNA was soon cloned and its function as an RNA-editing enzyme was recognized [4]. Interestingly, ADAR1 was also independently identified as an interferon-induced protein, and it was discovered that two isoforms are transcribed from the same gene with alternative splicing [5,6,7]. Two other members of this family, ADAR2 [8] and ADAR3 [9,10,11], were then identified by referencing ADAR1′s cDNA sequence information. While A-to-I RNA editing could be attributed to ADAR1 and ADAR2 in mammalian cells, no catalytic activity was detected for ADAR3 [12,13,14]. A-to-I RNA editing is a post-transcriptional process that converts selected adenosine (A) residuals to inosine (I) in the double-stranded regions of RNA transcripts [12,15,16,17,18]. Since inosine mimics guanosine (G) in Watson–Crick base pairing and during mRNA translation, A-to-I editing alters the RNA structure and changes the coding sequence of proteins [12,13,19,20]. Although a large number of editing sites have been identified, only a relatively small number of editing sites change protein coding; among such edited proteins are neuron receptors and ion channels [14,21,22,23]. However, these early examples of editing events still serve as the best illustrations for understanding the biological consequences of RNA editing. For example, the editing of the Q/R site in GluR-B mRNA by ADAR2 [24] dramatically changes the permeability of the ion channel of the AMP receptor. A-to-I RNA editing also modifies microRNA precursors and therefore impacts the biogenesis or shifts the targets of the corresponding miRNAs [25,26]. The mechanism and function of RNA editing in these traditional editing sites have been very well summarized in previous reviews [12,16,19,20,27,28]. To date, millions of editing sites have been identified or predicted using high-throughput methodology [29,30,31,32,33,34]. Most of the editing sites, however, were found to fall into non-coding regions [30,31,32,33,35]. Among these non-coding RNAs targets, the biological significance remains to be specified for most of the editing sites, although functions for some edited non-coding RNA have been identified within microRNAs [25,26,36,37] or in recognition sequences on the 3′UTR of certain mRNAs [38]. Recent studies have also shown that editing on the 3′UTR of cathepsin S mRNA (CTSS) enables the recruitment of the stabilizing RNA-binding protein human antigen R (HuR), therefore modifying its stability [39], and that editing affects pre-RNA splicing on smooth muscle cell marker mRNA [40]. However, how A-to-I RNA editing regulates innate immune response has not been well explained.

ADAR1 was originally thought to be the enzyme responsible for GluR-B mRNA editing [41,42] and through this function significantly affect neurological functions [43,44,45]. However, this important editing was subsequently attributed to ADAR2 [24]. ADAR1 indeed participates in the editing of many other sites; however, no significant biological function was linked to its editing sites that would explain its role in embryos [24,46], casting doubt on the significance of ADAR1 in RNA editing [14]. However, results from animal models with genetically disrupted ADAR1 expression showed that ADAR1 plays an indispensable role in embryonic development, since interruption of the ADAR1 allele leads to embryonic lethality with obvious defects in liver hematopoiesis and cell death [46,47,48].

Although this critical function for ADAR1 was demonstrated in knockout animal models more than a decade ago, the delineation of the mechanism by which it functions turned out to be challenging. In addition to its role in editing mRNA and microRNA precursors [25,38,47,49], ADAR1 has been shown to have editing-independent activities [26,50,51], participate in protein complex formation [25,52], and regulate RNA stability and translation efficiency [53,54,55]. Yet a specific RNA substrate, like GluR-B mRNA for ADAR2, has not been identified for ADAR1, and whether such a critical RNA molecule exists is uncertain. On the other hand, ADAR1 function might be mediated by the concurrent effects of numerous edited substrates, so that any single RNA molecule edited by ADAR1 would not exhibit significant consequences when viewed in isolation. Recent evidence supports the notion that in the condition of inappropriate RNA editing catalyzed by ADAR1, endogenous cellular RNA activates cytosolic RNA sensing signaling pathways, upregulates IFN production, and elicits innate immune responses.

## 2. ADAR1 and IFN Signaling

ADAR1^−/−^ embryos die around 11.5–12 days post coitum (d.p.c.) with substantial cell death in the liver [47,48]. A genome-wide transcriptome analysis of the microarray data from ADAR1^−/−^ embryonic liver hematopoietic stem cells revealed that ADAR1 gene deletion was strongly associated with a gene expression signature of type I and type II interferon-stimulated genes (ISGs) [56]. At the mRNA level, genes upregulated up to 300-fold include the transcription factors STAT1, STAT2, IRF1, IRF7, and IRF9; the GTPases Mx1 and Mx2; PKR; the 2′,5′-oligoadenylate synthases OAS1, OAS2, and OAS3; the ubiquitin-like modifiers ISG15 and ISG20; and the interferon-induced proteins Ifit1–Ifit3 [56]. Interestingly, among the dsRNA-induced gene expression of RNA binding proteins, only IFN-inducible genes were upregulated in ADAR1^−/−^ cells. Protein levels of IFN-α and IFN-β were also dramatically increased in the embryonic tissue, although type II IFN-γ was not detectable [56]. These findings implicated ADAR1 in the regulation of IFN and ISG in hematopoietic cells. How ADAR1 deficiency caused IFN expression that upregulated IFN signaling was not addressed in this early study. Nevertheless, the finding established a connection between ADAR1 and IFN signaling and eventually led to the discovery of the regulatory roles of ADAR1 in innate immune responses.

A link between ADAR1 and type I IFN production in human also came from observations in Aicardi–Goutières syndrome (AGS) [57], a severe autoimmune disease with early onset encephalopathy associated with a high level of IFN-α in the cerebrospinal fluid and upregulated ISG transcription [58,59,60,61]. A subtype of AGS was found to be associated with multiple mutations in the ADAR1 gene [57]. AGS is also associated with gene mutations of TREX1; RNASEH2A, 2B, and 2C; SAMHD1; and MDA-5 [59,62,63,64]. All the coded proteins from these mutated genes act on nucleic acids or sense nucleic acid signals in cells, indicating a connection between the increased IFN level and abnormal nucleic acid process. Considering that ADAR1 acts on dsRNA [12,13,65,66] and regulates IFN expression, it is possible that excessive IFN production in the neural tissue of AGS patients shares a mechanism with that in the ADAR1^−/−^ mouse model. In fact, we observed that inducible deletion of ADAR1 in newborn mice resulted in IFN-α and IFN-β gene expression in brain tissue [67]. It is known that A-to-I editing occurs to a much greater extent in human cells than in mice due to the high content of the repeat sequences in the human genome [68], such as the Alu element, which only exists in primates. Therefore, the pathogenesis of diseases caused by errors in RNA editing might be more pronounced in humans than in mice. This species difference might also partially explain why gene mutation rather than homozygous deletion of ADAR1 causes severe tissue injuries in AGS.

## 3. ADAR1 Suppresses the Sensing of Endogenous RNA by Cytosolic dsRNA Receptors

**Figure 1:**
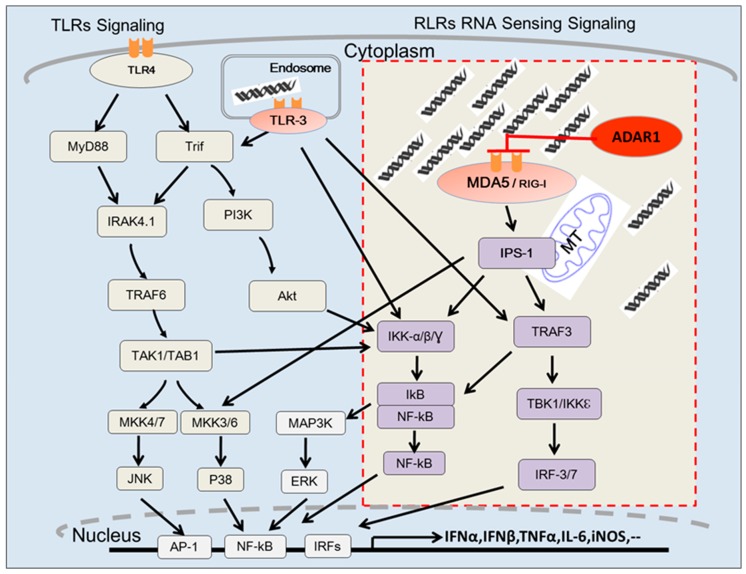
RAN sensing signaling pathways. Innate immune-active dsRNA is detected by either TLR3 or RIG-I like receptors (RLRs). While TLR3 detects extracellular RNAs, RLRs detect cytosolic RNA. Signal cascade from TLR3 to inflammatory cytokine gene expression include TRIF, IKKs and NF-kB. RLRs stimulation mainly leads to type I IFN production through IPS-1 (MAVS), TBK1/IKKε and IRFs. ADAR1 suppresses cytosolic RNA sensing in response to endogenous cellular RNAs by inhibition of the stimulation of RLRs by cytosolic RNA.

As an IFN-inducible protein, ADAR1 has long been believed to play critical roles in viral infection [5,69,70]. IFN-stimulated response element (ISRE) was found in the promoter region of the ADAR1 P150 isoform, which is responsible for IFN-induced ADAR1 expression [6,7]. However, until recently, the mechanism by which ADAR1 deficiency activates IFN signaling was not understood. Taking advantage of our inducible ADAR1 KO models [71,72], we first demonstrated that ADAR1 suppresses IFN signaling by preventing the detection of viral and cellular RNAs by cytosolic RNA receptors [67]. Deletion of ADAR1 in the cells was shown to dramatically increase type I IFN expression, including IFN-α and IFN-β. It is well established that microbial RNA stimulates type I IFN expression through RNA receptors including the endosome membrane anchored Toll-like receptor 3 (TLR3) [73,74,75] and cytosolic localized RNA receptors, RIG-I-like receptors (RLRs), which include RIG-I (retinoic-acid-inducible protein I) and MDA-5 (melanoma-differentiation-associated gene 5) [76,77]. After ruling out the involvement of TLR3, we found that downregulation of RIG-I or MDA-5 suppressed the impact of ADAR1 deletion on type I IFN expression [67]. RLRs detect cytoplasmic dsRNA and lead to activation of the protein kinases TBK1/IKKε through the adaptor protein mitochondrial antiviral-signaling protein (MAVS, also called IFN-β promoter stimulator-1, IPS-1), which in turn phosphorylates IRFs. Phosphorylated IRFs and NFκ-B form dimers and translocate to the nucleus to activate type I IFN gene expression [78,79,80,81] (Figure 1). Although our study was more focused on RIG-I, we found that MDA-5 knockdown also blocks the increase in type I IFN production caused by ADAR1 deletion [67]. This finding was soon confirmed by independent groups and reinforced by evidence from in vivo experiments that genetic deletion of MDA-5 or MAVS rescued the embryonic lethal phenotype of ADAR1 deficiency [82,83,84]. ADAR1^−/−^ embryos could not survive beyond 12.5 d.p.c. [47,48], but deletion of either the critical adaptor protein MAVS for RNA-sensing signaling or the RNA receptor MDA-5 rescued the embryos to birth, although postnatal death still occurred in some ADAR1 KO mice [82,83]. As expected, overexpressed ISGs were suppressed in the double KO mice. Collectively, all in vitro and in vivo data strongly support the conclusion that ADAR1 suppresses the cytosolic RNA-sensing signaling in response to endogenous cellular RNA.

Type I IFN is known to play critical roles in antiviral infection [85] and can induce death in infected cells or cancer cells in malignant diseases [86,87,88]. Upon binding to the cell membrane receptors IFNAR1/IFNAR2 and IFNGR1/IFNGR2, IFN triggers cell signaling through the transcription factor STAT 1 to induce expression of the ISGs [70,89]. It was thought that the robust upregulation of ISG expression in ADAR1^−/−^ mice contributed to embryonic death. However, a recent study found that deletion of the *Ifnar*1 gene (encoding the receptor for type I IFN) or STAT 1 did not prevent the lethality of ADAR1 deficiency [83]. Therefore, the increase in IFN signaling is unlikely the main reason for ADAR1^−/−^ embryonic death. This is also consistent with work showing that increased IFN can be tolerated [90]. The detailed mechanism responsible for the embryonic lethality resulting from ADAR1 deletion remains to be determined.

## 4. RNA Editing Activity of ADAR1 and the Innate Immune Response

The deamination activity of ADAR1 and ADAR2 converts adenosine to inosine in the double-stranded regions of RNA molecules, yielding inosine-containing RNA in cells [12,19,20]. Vitali and Scadden showed that chemically synthesized short dsRNAs (20 bps) containing inosine–uridine pairs (I-U dsRNA) suppressed ISG expression in HELA cells stimulated with poly I:C transfected into the cytoplasm, or expressed exogenous RNA with a potential dsRNA structure (Fluc mRNA) [91]. The inhibitory effect of the I-U RNA was independent of the short RNA sequence, but a minimum number of three inosines (I-U pairs), located in the middle of the 20-base pair fragment, was required for sufficient suppression of ISG expression. Interestingly, the I-U RNA bound to the cytosolic RNA receptors MDA5 and RIG-I and inhibited IRF3 activation. This finding demonstrated that I-U dsRNA may suppress ISG induction in response to long dsRNA through binding to the RLRs. However, whether the converse is true (increased ISG expression in ADAR1-deficient cells was due to lack of I-U RNAs) was not known. It is important to point out that the three consecutively positioned I-U pairs in the middle of the 20 bp dsRNA fragment required for ISG suppression are not likely to exist or are very rare in normal cells. The efficiency of A-to-I conversion catalyzed by ADARs on most editing sites is usually low, and RNA molecules with consecutively positioned adenosines that are highly edited have not been reported. In addition, ISG expression stimulated by transfected exogenous long dsRNA, which mimics viral infection, does not represent the immune active endogenous RNAs. However, a recent study carried out by Liddicoat and colleagues provided convincing evidence that RNA editing by ADAR1 is absolutely required for the suppression of IFN production [84]. In a genetically mutated mouse model, a single nucleotide mutation in the catalytic domain of ADAR1 abolished its RNA editing activity but preserved the RNA binding and z-DNA binding domains of full length ADAR1 (a single-nucleotide mutation knock-in, KI, mouse model). These mice exhibited a very similar phenotype of embryonic lethality as was observed in ADAR1 KO mice lacking the entire protein. Upregulated ISG expression was also observed in the cells of the KI mice. When bred with MDA-5 KO mouse, MDA-5 deletion not only blocked the ISG expression but also rescued the ADAR1 KI mice to the adult stage [84]. In combination, these findings support a theory that ADAR1 edits cellular RNA transcripts; through edited RNA, ADAR1 prevents activation of the cytosolic RNA-sensing pathways. Based on this finding, it has been hypothesized that RNA editing by ADAR1 marks dsRNA as “self” [92] and ADAR1 editing distinguishes the self from the non-self RNA [93]. However, due to the low efficiency of ADAR1-catalyzed RNA editing, a large portion of the “self” RNA is not edited. To prove the hypothesis, a particular edited cellular RNA or a group of such RNA molecules needs to be demonstrated to be sufficient to silence the innate immune response. In addition, the suppression of the edited RNA on RNA sensing may not be specific for endogenous RNA and also suppresses the viral RNA. How ADAR1 distinguish “self” from “non-self” will need to be explained.

## 5. Cytosolic RNA Receptors Responding to Cellular “Self” RNA of ADAR1 Substrate

Both RIG-I and MDA5 are cytosolic RNA receptors that detect microbial RNAs and trigger an innate immune response through IFN signaling [78,80,81]. However only MDA-5 deletion, not RIG-I deletion, mitigates the lethality of ADAR1 deficiency [82,84], indicating that only MDA-5 is involved in RNA sensing in ADAR1 KO cells. While both RIG-I and MDA5 detect viral RNAs, these receptors exhibited selective recognition of their ligands [80,94]. MDA5 recognizes poly I:C and RIG-I detects in vitro transcribed dsRNA, as shown by studies in the specific KO mouse models [94]. They may also respond to different endogenous RNA in ADAR1^−/−^ embryonic cells. While both RIG-I^−/−^ and MDA5^−/−^ mice were highly susceptible to infection with respective RNA viruses, MDA5^−/−^ mice did not show obvious abnormalities in noninfectious conditions. In contrast, most RIG-I^−/−^ embryos died at embryonic days 12.5 to 14.0; a few mice survived to birth but died within three weeks [81]. RIG-I seems to play more important roles in embryonic development compared to MDA-5. Embryonic death of RIG-I^−/−^ and ADAR1^−/−^ embryos occurred at very similar time points. A combination of deletion of both ADAR1 and MDA-5 genes may result in a complicated disruption of cellular function besides RNA sensing. Although RIG-I deletion did not rescue the lethality of ADAR1^−/−^ embryos, it did not rule out the possibility that ADAR1 regulates RIG-I initiated RNA-sensing signaling in certain pathological conditions. In experiments with cell lines infected with virus or hypoxemia treated adult mouse hepatocytes, we observed that downregulation of RIG-I suppressed type I IFN production caused by ADAR1 deficiency [67,95,96]. It is possible that endogenous RNAs differentially stimulate RIG-I or MDA-5 to active the RNA sensing pathway in ADAR1-deficient embryos, adult tissue, or specific cell types.

## 6. ADAR1 Edited Cellular dsRNA and Innate Immune Response

Identification of the editing site(s) in potential RNA substrate is the goal of RNA editing studies. Unbiased screening methods, including chemical methods [97,98,99], cyber analysis of the large databases of expressed sequence tags [30,31,32,33], and genome-wide RNA-seq with bioinformatics analysis [29,100,101,102], were developed and applied to systemic identification of RNA editing sites. Depending on the method and screening criteria and species origin of the RNA, the total numbers of editing sites identified/predicted fall within a wide range from thousands to hundreds of millions. In human cells, it is now known that most of the RNA editing sites are in non-coding regions, especially the 3′UTRs. Consistent with the requirement for double-stranded RNA structure for editing, most editing sites were found in short interspersed nuclear elements (SINEs), especially the short Alu repetitive sequences, which compose more than 10% of the human genome [103]. Alu elements closely situated and reversely orientated in RNA transcripts potentially form dsRNA structures that serve as the editing targets. Cell type-specific editing sites and clustered patterns of editing have been shown by studies and the editing pattern may change under different pathologic conditions [84,104,105]. Besides editing on the 3′UTR, which affects mRNA stability, translation efficiency, and microRNA recognition, the function of most editing sites in non-coding regions has not yet been determined. Suppression of the cytosolic RNA-sensing pathway might be one of the major functions of edited non-coding RNAs. As discussed above, inosine-containing RNAs yielded by RNA editing may change the RNA structure that inhibits RNA receptors. However, the presence of inosine or mismatched base pairs in dsRNA does not guarantee its inhibitory activity. For example, Poly I:C contains 100% inosine in one of its double strands, but it stimulates rather than suppresses type I IFN production. Short dsRNA containing G-C pairs (vs. I-U pairs) did not have an inhibitory effect on the IFN-inducing pathway [91]. Thus, the inhibitory effect of the edited RNA must meet certain criteria. In addition, editing efficiency on most editing sites is not very high (it has been shown to be as low as <1% by many high-throughput methods including RNA-seq bioinformatics analysis). For a dsRNA molecule with multiple editing sites in it, many editing patterns may be generated with variant editing at different sites, while most RNA transcripts from the same gene remain unedited. The specific editing patterns necessary to silence the RNA sensing pathway remains to be determined.

**Figure 2:**
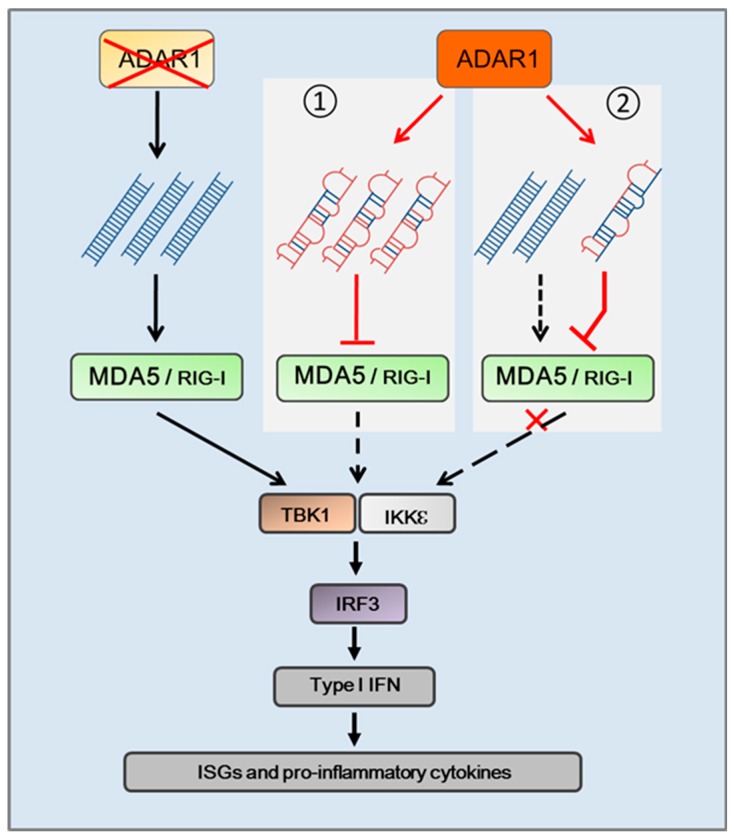
Hypothesis of the mechanism by which cellular endogenous RNA edited by ADAR1 silences cytosolic RNA sensing signaling pathway. In the condition of ADAR1 deficiency, endogenous dsRNA stimulates cytosolic RNA receptors to elicit innate immune response. RNA editing by ADAR1 changes the dsRNA structures by introducing mismatched I-U base pairs into the RNA transcript, which replace A-U pairs in perfect dsRNA. If editing is sufficient to nullify all immune active RNAs, the corresponding receptors remain unstimulated, as shown in box 1. If RNA editing efficiency is low, the edited dsRNA will need to be high capacity to inhibit the stimulation from the unedited immune active RNAs, as shown in box 2.

Under biological conditions, it appears that the activity of the innate immune system is kept in check through ADAR1 editing of selected RNA transcripts. In such a condition the cytosolic RNA-sensing pathway of the innate immune system does not respond to endogenous cellular RNAs. The silent RNA-sensing pathway might be maintained through two different mechanisms (Figure 2). First, while dsRNA structures are formed in the nascent RNA transcripts, which are capable of activating the innate immune response, ADAR1 converts A-U pairs to I-U pairs, eradicating all the immune active dsRNA and thereby preventing the autoimmune reaction. If this is true, RNA editing marking cellular RNA as “self” could be a valid concept, as proposed [92]. Second, it is possible that the small quantity of inosine-containing RNA generated by RNA editing is sufficient to prevent the activation of the RNA receptors. It has been proposed that inosine RNA might prevent MDA-5 oligomerization, therefore preventing down-stream signaling [84]. In this case, the I-U-containing RNA should be highly efficient in the inhibition of RNA sensing, and may not be limited to self RNA stimulation. Here, edited RNA might also inhibit viral RNA stimulation, especially when ADAR1 is induced during infections. In any case, it will be necessary to find a particular RNA transcript in cells to demonstrate that editing of this RNA is sufficient for suppression of the RNA sensing signaling. This remains a significant challenge.

There are many questions that need to be answered for a better understanding of the mechanism by which ADAR1 suppresses the innate immune response through edited RNAs. For example, why does only ADAR1 and not ADAR2 deficiency cause IFN production? Although ADAR1 and ADAR2 show some specificity for RNA substrates, many editing sites are catalyzed by both proteins [106]. It has also been shown that the ADAR1 isoform P150 is more critical than P110 in embryonic development and in regulation of IFN production [50,82]. P110 is a nucleic protein, while P150 is found mainly in the cytoplasm. The different distribution of these two isoforms might partially account for their different functions. However, ADAR-catalyzed RNA editing is believed to mainly occur in the nucleus before introns are spliced out; P110 is also the major isoform expressed in many tissues, including neural tissue, where A-to-I RNA editing was first identified to play a critical role. P110 should be the major enzyme for ADAR1′s editing function. Why RNA must be edited by cellular P150 to suppress the IFN induction has not been well explained.

## 7. Conclusions

In summary, the RNA editing enzyme ADAR1 plays a critical role in the regulation of innate immune activation through the suppression of endogenous RNA sensing. ADAR1 deficiency causes sterile inflammation and tissue injury and is implicated in many diseases. The catalytic activity of ADAR1 that converts adenosine to inosine in the dsRNA region of RNA transcripts was shown to be necessary for its inhibitory function in INF production. Specific RNA editing by ADAR1, not ADAR2, suppresses the immune response to endogenous RNA. However, RNA editing efficiency is usually low on most editing sites. How edited RNA suppresses the RNA-sensing pathway is not yet fully understood.
